# Recent atmospheric drying in Siberia is not unprecedented over the last 1,500 years

**DOI:** 10.1038/s41598-020-71656-w

**Published:** 2020-09-14

**Authors:** O. V. Churakova Sidorova, C. Corona, M. V. Fonti, S. Guillet, M. Saurer, R. T. W. Siegwolf, M. Stoffel, E. A. Vaganov

**Affiliations:** 1 Siberian Federal University, Svobodny pr. 79, Krasnoyarsk, Russian Federation 660041; 2 Institute for Environmental Sciences, University of Geneva, 66 Bvd Carl Vogt, 1205 Geneva, Switzerland; 3 Geolab, UMR 6042 CNRS, Université Clermont-Auvergne (UCA), 4 rue Ledru, 63057 Clermont-Ferrand, France; 4 Swiss Federal Institute for Forest, Snow and Landscape Research WSL, Zürcherstrasse 111, 8903 Birmensdorf, Switzerland; 5 Dendrolab.Ch, Department of Earth Sciences, University of Geneva, Rue des Maraîchers 13, 1205 Geneva, Switzerland; 6 Department F.-A. Forel for Environmental and Aquatic Sciences, University of Geneva, 66 Bvd Carl Vogt, 1205 Geneva, Switzerland; 7 V.N. Sukachev Institute of Forest SB RAS, Federal Research Center “Krasnoyarsk Science Center SB RAS”, 50/28 Akademgorodok, Krasnoyarsk, Russian Federation 660036

**Keywords:** Climate sciences, Ecology, Environmental sciences

## Abstract

Newly developed millennial δ^13^C larch tree-ring chronology from Siberia allows reconstruction of summer (July) vapor pressure deficit (VPD) changes in a temperature-limited environment. VPD increased recently, but does not yet exceed the maximum values reconstructed during the Medieval Warm Anomaly. The most humid conditions in the Siberian North were recorded in the Early Medieval Period and during the Little Ice Age. Increasing VPD under elevated air temperature affects the hydrology of these sensitive ecosystems by greater evapotranspiration rates. Further VPD increases will significantly affect Siberian forests most likely leading to drought and forest mortality even under additional access of thawed permafrost water. Adaptation strategies are needed for Siberian forest ecosystems to protect them in a warming world.

## Introduction

Precipitation and permafrost-thawed water during the summer are crucial for trees growing in regions with severe temperature limitations and predominantly continental climate conditions^1–4^. Although precipitation is low in the Siberian North, due to low temperatures, water loss is not yet as large as observed in European forest ecosystems^5^. However, increasing surface air temperature^3,6,7^ over the period 1950–2008 driven by the anthropogenic CO_2_ increase, may also lead to drought stress accordingly^8–12^. By enhancing the atmospheric vapor pressure deficit (VPD)^13,14^, even in remote subarctic regions, increased evapotranspiration impacts in particular the tree’s water relation. Ongoing anthropogenic warming already has significant negative effects on Siberian forest ecosystems, leading to increased permafrost degradation and CO_2_ emissions^15^. Along with temperature increases, increasing VPD^14^ has been shown to result in low stomatal conductance^3^, thereby responding to water shortage in trees—even in regions affected by permafrost^3^. While water and nutrient supply for plants in the Siberian North predominantly depends on freeze–thaw processes in the active soil layer^16,17^, wildfire-induced changes can also significantly affect the active soil layer depth and seasonal dynamics with long-term consequences for carbon, nutrient and water balance of the ecosystem^8,18,19^.

Climate models project drastic changes in climate even under the optimistic RCP 2.6 scenario for the Siberian regions for the period 2081–2100 relative to 1986–2005: (1) a strong temperature increase and reduced moisture availability leading to forest die-back and northward shift of boreal ecosystems^20^, and (2) a strong temperature increase up to 6 °C with increased precipitation bringing more frequent flooding events^21^. Based on these projections by the climatic models it becomes clear that the recent rapid climatic warming propose a heterogeneous changes in precipitation patterns.

To reduce such uncertainties in climatic models, knowledge about past climate variability is crucial and can be provided by annually resolved tree-ring records^22^. Tree-ring width, latewood density and wood anatomical parameters record mainly summer air temperature signals for the Eurasian subarctic^6,7,23–25^, while little is known about the past hydro-climatic regime (e.g., precipitation, vapor pressure deficit, relative air humidity)^25^. Additional paleoclimatic proxies like stable carbon isotopes in tree rings should therefore be considered, which can provide information not only about temperature but also about moisture changes in the temperature limited environment^25,26^. The carbon isotope ratio (^13^C/^12^C) is impacted by the same environmental variables as photosynthetic rate (A) and stomatal conductance (g_s_)^27^, which can be an ideal tracer of moisture changes. Low δ^13^C values usually indicate high air humidity (low VPD) and good water supply (usually resulting in high *g*_*s*_ values) in contrast to higher (less negative) δ^13^C values by indicating drought (low *g*_*s*_).

Stable isotopes have been used to derive reconstructions for subarctic regions of millennial air temperatures^28^, irradiance^29–31^, and hydroclimate changes^32–34^. A recent review by the Past Global Changes (PAGES) Hydro2K Consortium^35^ compared climatic models and different paleoclimatic archives^35,36^ to estimate hydroclimate variability and change over the Common Era. However, studies of stable isotope in tree-rings from remote Siberian regions are still rare and urgently needed, because the impact of anthropogenic activity is still relatively low in these regions, and started much later^2,15,26^ than in industrialized and highly populated areas^20^.

To reveal the impact of recent climate changes on Siberian forests we used the imprinted isotope signal from living and dead larch trees stored in permafrost as a climate proxy to assess pre-industrial (CE 516–1,850) and anthropogenic (CE 1,850–2004) changes in stable carbon (δ^13^C) isotopes, to reconstruct a vapour pressure deficit (VPD) over the past ~ 1,500 years.

## Results

### Development of the annual 1,448-year δ^13^C chronology

**Figure 1:**
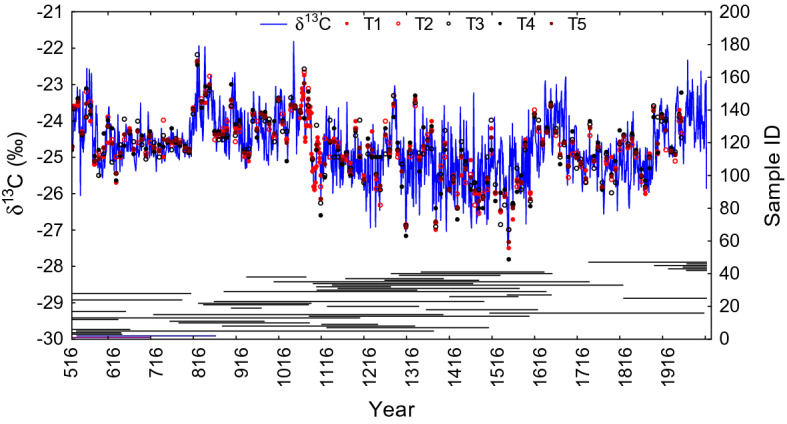
A 1,489-year δ^13^C chronology from larch tree-ring cellulose was constructed based on annually cross-dated samples (n = 48). Individual tree measurements used to verify the range of variability of each tenth-year (T1, T2, T3, T4 and T5) are marked with circles of different colors.

For the first time for the Eurasian subarctic, a continuous δ^13^C chronology was constructed based on larch (*Larix cajanderi* Mayr.) tree-ring cellulose samples with annual temporal resolution for the period from CE 516 to 2004 (Fig. 1).

Abrupt shifts from 0.8‰ up to 3.2 ‰ and an increase in the standard deviation (SD) from 0.2 to 0.9 of mean carbon isotope values are observed for the periods CE 780–810 and CE 1060–1090 when compared to the climate norm (i.e. periods of 30 years before and 30 years after the shifts) (Fig. 1, Table S3a).

High δ^13^C values were revealed mainly for the Medieval Climate Anomaly (MCA, CE 800–1070) period with a mean value of − 23.9‰ and a maximum value of − 21.8‰ and for the Recent Warming Period (RP, 1950–2004) with a mean value − 23.9‰ and a maximum value of − 22.3‰. Standard deviation of the MCA (SD = 0.64) was similar to the RP (SD = 0.66) (Table S3b).

The lowest δ^13^C values were detected during the Early Medieval Period (EMP, CE 516–799) and during the Little Ice Age (LIA, CE 1450–1850) periods, where minimal values were − 26.05‰ (mean − 24.54‰) and − 27.49‰ (mean − 25.03‰), respectively (Table S3b). A higher standard deviation was found for the LIA (SD = 0.8) as compared to the EMP (SD = 0.5).

### Reconstructed summer vapor pressure deficit anomalies over the past 1,489 years

The correlation and response function analyses showed that the variations of δ^13^C values were driven by July VPD. Regression analyses applied for the calibration (1959–1981) and verification (1982–2004) periods, and for the whole period of observations (1959–2004) showed significant (*p* < 0.01) and stable statistical relationships over the full length of the study period (Fig. S2a, Table S2). The good statistical agreement can be underlined further with the results obtained with the verification test [R^2^ and reduction of error (RE)] for the calibration period, as well as R^2^ and coefficient of efficiency (CE) values for the verification period (see “Methods” section and Table S2). Almost 34% of the variance in δ^13^C can be explained by July VPD with RE = 0.32 and CE = 0.49. The decadal variability are still robustly explained by July VPD for the period from 1959 to 2004, r = − 0.30, *p* = 0.0001 after the de-trending procedure, demonstrating the robustness of the reconstruction.

**Figure 2:**
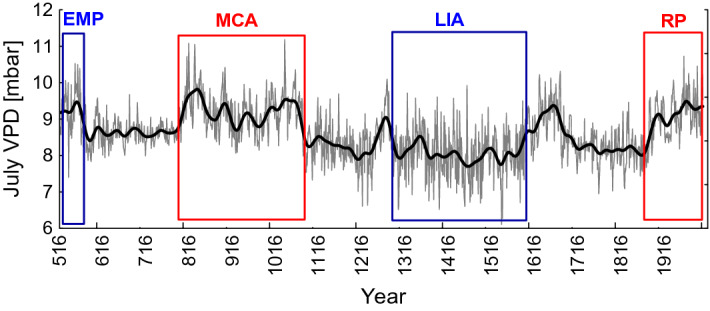
Annually resolved (grey line) and smoothed by a 41-year Hamming window (black line) July VPD reconstruction over the period from 516 to 2004. The Early Medieval Period (EMP), Medieval Climate Anomaly (MCA), Little Ice Age (LIA) and the Recent Period (RP) for northeastern Yakutia (C-IND) are indicated in blue and red rectangles.

Based on the regression model, July VPD reconstruction for the period from CE 516 to 2004 was obtained. The lowest value relative to the mean value of the entire analyzed period (8.6 mbar) was found for 1538 (− 3.4 σ; 6.1 mbar). The highest simulated VPD value in the past 1,489 years was in 1,036  (≈ 11.2 mbar or + 3.5σ), while a greater number of negative anomalies over the past periods were recorded during the LIA (− 3.4σ) (Fig. 2).

Minimum values of the negative simulated VPD anomalies (humid) (− 3.4σ; SD = 0.9) were revealed for the period CE 1516–1616. For the periods, CE 1116–1616 and CE 1716–1916 anomalies were within the range of − 2.09 σ to − 2.88 σ with the SD from 0.5 to 0.9, respectively to both periods (Table S4).

Maximum values of the positive (dry) simulated VPD anomalies (+ 3.5σ) were revealed for the MCA (CE 800–1,070) and the RP (CE 1901–2004) (+ 2.9σ) periods, highlighting extreme anomalies during the medieval period compared to the recent period. Less pronounced increasing July VPD trends were detected for the periods CE 616–716, 1116–1216, 1316–1416, and 1416–1616.

### Spatio-temporal patterns of evapotranspiration variability over the twentieth century

**Figure 3:**
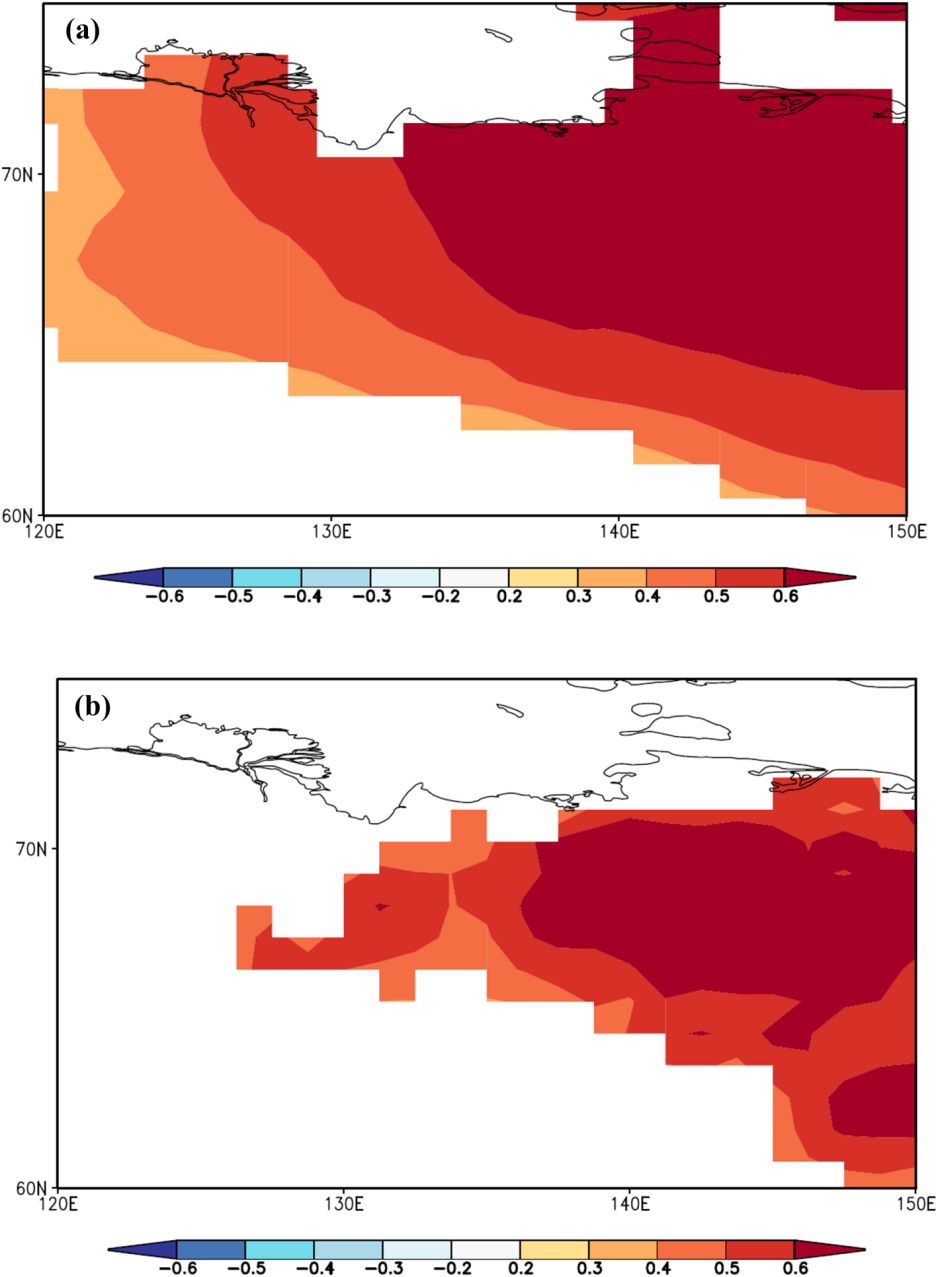
Spatial correlation maps of VPD reconstructed data versus gridded maximum July temperature **(a)** and July evapotranspiration **(b)** for the available period 1979–2004, respectively.

Reconstructed July VPD is highly correlated with the CRU gridded July maximum air temperature (Fig. 3a) and July evapotranspiration (Fig. 3b) data is available for the period from CE 1979 to 2004 over a large region of Northern Eurasia. This spatial correlation analysis shows clear indications of drought conditions under July air temperature increase. A few studies^37,38^ showed a drastic VPD increase and a relative humidity decrease after the 2000s for other sites worldwide.

Data of the SPEI Global Drought Monitor showed a significant negative correlation (r = − 0.48; *p* < 0.05) with our VPD reconstruction indicating drought conditions in the study region for the period with available data for the July evapotranspiration CE 1979–2004 (Fig. 3b).

## Discussion and conclusion

Reconstructed July VPD showed highest values during the Medieval Climate Anomaly period (800–1070) in the Siberian North compared to the period (1870–2004). This finding is similar to reconstructions of June–July air temperatures for this region using tree-ring widths from the same trees^39^. The recent and drastic thawing of permafrost under the ongoing global temperature increase at the study site^3^ has analogues back in time showing a similar pattern during the MCA. We assume that the observed shift in C-IND around 1,060-ies can be considered as a consequence of the medieval warming on a previous episode of a permafrost temperature increase and thawing during the early second millennium. Temperature increases and a reduction of precipitation can result in permafrost degradation^40,41^, which impact of important hydro-climatic factors such as the interplay between water source, soil moisture and soil thaw depth influence tree growth under permafrost conditions. Dynamics of soil moisture and soil temperature are dominant factors and can thus influence carbon isotope ratios significantly at our study region^3^. Changes in environmental conditions are thus the likely cause of the changes we observe in the reconstruction, as we cannot attribute these changes to potential biases in sample coverage or the inclusion of young(er) trees.

Dry conditions during the MCA are recorded in tree rings from Fennoscandia and Arctic Canada^29,42^. However, most likely wet conditions during the LIA were recorded in pollen records from the Canadian Arctic^43^. Our findings suggest a trend towards higher simulated VPD (almost 10%) across the entire period (516–2004 CE) compared to the global average of 1%^14^. The drastic thawing of permafrost in the subarctic forest and reduction of precipitation with increasing evaporative demand^4,13,44^ could increase the risk of forest decline and mortality at these sites in the (near) future.

The reconstruction of July VPD showed that MCA was not only warm^25,45^ but also was dry in northeastern Yakutia and started earlier (since 800 CE) compared to the western part of the Eurasia subarctic^31^ (Fig. 3). Positive reconstructed VPD extremes were recorded in annual δ^13^C stable isotope chronology obtained from long-living larch trees over the Medieval Climate Anomaly (MCA) and the recent periods, while humid conditions were recorded during early first millennia and the Little Ice Age. Helama et al.^29^ reported extremely dry MCA compared to LIA in the post-industrial period (1850–present)^42^.

Negative anomalies of δ^13^C values are associated with negative VPD anomalies and probably result from less isotopic discrimination driven by high stomatal conductance values and moderate net photosynthesis. The RP is characterized by increasing temperature and development of drought conditions with increasing VPD of ca. 0.8 mbar (CE 1950–2004) compared to the past (CE 516–1949).

Significantly correlated spatial distribution patterns of maximum July air temperature and July evapotranspiration with the reconstructed VPD chronology are further suggesting the development of drought conditions in the permafrost region under investigation. We thus conclude that increased VPD under raised air temperature and evapotranspiration may trigger stomatal closure to minimize water loss^14,27^. Furthermore, trees most likely adapt their root system to changing environmental conditions or will ultimately die off due to water shortage. Further eco-hydrological studies are needed for this unique Siberian region to develop adaptation strategies for Siberian forest ecosystems to protect them in a warming world.

## Materials and methods

### Study site

The study site is situated in northeastern Yakutia, Indigirka (IND, 69° N, 148° E, 160–340 m.a.s.l.), Sakha Republic (Fig. S1a), Russia. This region is characterized by extra-continental climate conditions. According to the instrumental data from the Chokurdach weather station (62° N, 147° E, 61 m.a.s.l.) for the available period from 1959 to 2004 the mean January air temperature is—34 °C, while July is + 15 °C. The winter season lasts from the end of August to end of May with a very short growing season of between 50 and 70 days. The sum of annual precipitation is only 205 mm. The lower part of the soil profile is characterized by continuous low-temperature permafrost that is 500–650 m thick and has a temperature range of − 10 to − 12 °C^46^. The seasonal thaw depth of permafrost is not more than 30–50 cm, reaching maximum thaw depth (up to 70 cm) in July^3^. Soil moisture in the upper layers decreases during the summer season^41^. Soil dryness in July and August depends on the amount of precipitation during these months. The soil temperature at a depth of 20 cm is still below freezing point at the end of June, but may increase up to + 4 °C in July according to the daily measurements by Fyodorov-Davydov et al.^46^.

Relict wood samples and tree cores from living larch trees (*Larix cajanderi* Mayr.) were collected from 200 to 350 m.a.s.l. near At-Khaya mountain (69° 22–24′ N, 148° 25–35′ E) (Fig. S1a). A typical plot for sampling from old living as well as relict wood is located at the upper slope of the south-west exposition. It is a sloping upper terrace, which smoothly goes to the plane spur of watershed ridge. The middle part of the slope (250–300 m a.s.l.) is taken by scree laid by large rock formations. Larch open forests with tree layer density to 0.3 are presented by uneven aged stands with a sparse shrubby layer and prevailing tundra vegetation in the above-ground layer. Fresh habitats with mosaic soil are covered by large rock formations. The cobble-loamy mountain tundra frozen soils are mainly present here. The lichen-moss cover is poorly developed. Lichen and yernik series of forest type groups with visual differentiation of tree layer in morphometric parameters dominate here. This is an old generation formed at the boundary of the first and second millennia as well as a young generation formed in the 1,930–1,950ies. The old generation proportion is 0.1–0.2 of 1; diameter at breast height (DBH) is 26 cm; mean tree height is 6.0 m up to 9.0 m; trees show deep cracks in the bark; flat crown; more than 50% of trees have dry tops and stem rot. The young generation and regrowth dominate in open forest at the present upper larch timberline (200–350 m a.s.l.). Mean tree height is 5–6 m, DBH is 6 cm, trees have well-developed crowing. A great number of dry stems of different safety degree with the ratio of dead and living trees 1.5:1.0 up to (2.0:1.0) distinguishes the sparse tree layer. Two thirds of dead standing trees are due to windfall. The oldest living trees greater than 800 years old (Fig. S1b) are represented by the generation formed during the “Little Climatic Optimum” of the Holocene. Most of the dead trees are sphagnumised and fallen represent the tree generations formed in the first half of the current millennium (Fig. S1c). Only well preserved and visually healthy old trees were selected for the stable isotope analysis. Trunks of the subfossil wood were well stored on the ground surface due to the prevailing permafrost. Good sample preservation allowed the use of these samples for building the long-term tree-ring width, latewood density, and wood anatomy^2,25^ chronologies.

### Sample preparation

Larch stem discs and tree cores were selected from the available material, which was collected during several expeditions between 1997 and 2004 (Table S1)^45,47^.

Subsamples were selected based on previously calendar dated tree-ring width series and later on were cross-dated using the reference tree-ring width chronology^45,47^. All 48 selected subsamples needed for the construction of new stable carbon isotope chronology were re-measured and cross-dated using the standard dendrochronological Software (TSAP, DPL, ARSTAN)^48^.

Cross-dated subsamples were split for each annual ring separately with the scalpel. Average age of trees used for the carbon isotope analysis was 308 years. First 50 years of the juvenile stage^49^ were excluded from the analyzed wood samples. Then, each annual ring of individual or pooled wood sample was enclosed into a filter bag and α-cellulose extraction was performed according to the method described by Loader et al.^50^ and Boettger et al.^51^. Samples were prepared for each individual year and each larch tree separately for the period from 1945 to 2004. However, small sample size resulted in the need to pool material, i.e. annual rings from four trees were milled together for all of the other periods. To check coherence and offset between pooled tree-ring cellulose samples we measured trees individually for every 10th year (Fig. 1) according to Boettger et al.^52^. Expressed population signal (EPS)^52^ between δ^13^C chronologies of four individual trees is 0.95 for raw and 0.90 for atmospheric corrected according δ^13^C atmospheric CO_2_ data for the period from 1948 to 2004.

### Stable carbon isotope analysis

Stable carbon isotope analysis was performed within several research projects during the period from 2005 to 2017 at the stable isotope facility of the Paul Scherrer Institute, Villigen, Switzerland. Samples (0.2–0.3 mg) were weighed into tin capsules for the analysis of the ^13^C/^12^C using an isotope ratio mass spectrometer delta-S (Finnigan MAT, Bremen, Germany) linked to two elemental analyzers (EA-1110 Carlo Erba, Italy) via a variable open split interface (CONFLO-II, Finnigan MAT, Bremen, Germany). The ^13^C/^12^C was determined by combustion under excess oxygen at a reactor temperature of 1020 °C, operating in continuous flow mode.

Samples for the periods CE 550–910, 995–1250, 1280–1630, 1670–1795, and 1830–1895 were analyzed with a vario PYRO cube (Elementar, Hanau, Germany) via thermal decomposition at 1,450 °C and conversion to CO under O_2_ exclusion in helium^53^. This system was linked to a IRMS (Delta plus XP, Thermo Finnigan, Bremen, Germany).

Both systems the EA-IRMS and the PYRO cube yielded very similar precisions (± 0.2‰) and the values from the two instruments were in high agreements (better than the measurement precisions)^54^.

The isotopic values were expressed in the delta notation relative to the international standard in the Eq. (1):

1 $$ {\text{d}}_{{{\text{sample}}}} \left(\permil \right)_{{}} = \, \left( {{\text{R}}_{{{\text{sample}}}} /{\text{ R}}_{{{\text{standard}}}} - { 1}} \right) \cdot {1}000 $$

where R_sample_ is the molar fraction of ^13^C/^12^C of the sample and R_standard_, of the Vienna Pee Dee Belemnite (VPDB) standard. The precision of measurements is σ ± 0.1‰.

As the pyrolysis method via PYRO cube involves a small contribution of carbon from the reactor filling to the measuring gas, this needs to be corrected as proposed by Woodley et al.^53^ and further modified by Weigt et al.^54^, where δ^13^C_corrected_ = 1.1142 δ^13^C_raw_ + 1.4504. This correction equation is established by measuring a subset of samples via combustion with the elemental analyser. Additional quality control standards are used in each analysis run to test the stability and reproducibility of the system.

The δ^13^C data of cellulose for all years after 1,800 were corrected for the Suess effect (decline of the ^13^C/^12^C ratio of atmospheric CO_2_) using δ^13^C values of atmospheric CO_2_ obtained from the South Pole ice core and the Mauna Loa Observatory, Hawaii^55^ (https://www.cmdl.noaa.gov./info/ftpdata.html). This correction is necessary because the emission from fossil fuel combustion and biomass burning have resulted in decreasing δ^13^C values of atmospheric CO_2_. De-trending procedure was applied for the δ^13^C isotope chronology for comparison with climate data to check if decadal variability are still robustly explained by climate parameters.

### Climate data

Response function analysis was applied using Statistica 13.3 Software to evaluate the climate response of the Yakutia δ^13^C chronology against temperature, precipitation and vapor pressure deficit data available from the Chokhurdach weather station (62° N, 147° E, 61 m a.s.l., https://aisori.meteo.ru/ClimateR) for the period 1959–2004. Gridded July temperature and evaporation data were used (https://climexp.knmi.nl/correlate.cgi) to correlate the reconstructed July VPD and present distribution of the signal along the spatial scale.

The Standardised Precipitation-Evapotranspiration Index (SPEI)^56^ was computed (https://spei.csic.es/) for the period 1979–2004 to reveal and estimate drought conditions with a gridded net 1° × 1° spatial resolution and a monthly time resolution (available under the Open Database License https://opendatacommons.org/licenses/odbl/1.0/).

### Statistical characteristics

The δ^13^C tree-ring cellulose chronology (C-IND) was regressed against VPD instrumental data (1959–2004). C-IND shows significant correlation with VPD data, with the highest correlation being observed with the month of July (r = 0.49) (Table S2). Split-period calibration/verification statistics such as Pearson correlation coefficient (R), reduction of error (RE), coefficient of efficiency (CE), and Durbin–Watson statistics (DW), coefficient of synchronicity (K_s_)^57^ were computed to test the robustness of the transfer function (Fig. S2, Table S2). Calibration and validation statistics are illustrated with their 2.5 and 97.5 percentiles and the reconstruction is given with its 95%-confidence intervals. Kolmagorov-Smirnov test for level of significant was applied.

Trends were calculated as the slope of the linear regression from 1959 to 2016 CE for the δ^13^C versus VPD.

A new developed July VPD reconstruction derived from isotopic composition of δ^13^C in tree-ring cellulose and earlier obtained June-July air temperature reconstruction from tree-ring width for northeastern Yakutia^25,26,45^ were analyzed for climatic anomalies in comparison (Table S3). Normalized (z-score) data versus number of observations were calculated for the Early Medieval Period (EMP) 516–799, Medieval Climate Anomaly (MCA) 800–1070; Little Ice Age (LIA) 1450–1850^20^, and the Recent Period (RP)^20^ 1870–2004. Anomalies are shown relative to each analysed period from the mean and standard deviation over the 1489 years. Deviations ≥  ± 3σ from zero were considered as extreme anomalies, ≥  ± 2σ as anomalies.

## Supplementary information

Supplementary Information.

## Data Availability

The datasets generated during and/or analysed during the current study are available from the corresponding author on reasonable request.
