# Supplementary material for: Recent atmospheric drying in Siberia is not unprecedented over the last 1,500 years
Source: Sci Rep. 2020 Sep 14;10:15024. doi: 10.1038/s41598-020-71656-w (PMC7490406; doi:10.1038/s41598-020-71656-w)
Supplement: Supplementary file 1 — Supplementary Information. [file 41598_2020_71656_MOESM1_ESM.docx]

**Title: Recent atmospheric drying in Siberia is not unprecedented over the last 1500 years**

**Authors:** O.V. Churakova (Sidorova)^1,2,3*^, **C. Corona**^4^**,** M.V. Fonti^1,2^, **S. Guillet**^3^**, M. Saurer**^2^**,** R.T.W Siegwolf^2^**,** M. Stoffel^3,5,6^, **E.A. Vaganov**^1,7^

^1^Siberian Federal University, Krasnoyarsk, Svobodniy pr. 79, 660041 Russian Federation

^2^Swiss Federal Institute for Forest, Snow and Landscape Research WSL, Zürcherstrasse 111 8903 Birmensdorf, Switzerland

^3^Institute for Environmental Sciences, University of Geneva, 66 Bvd Carl Vogt, 1205 Geneva, Switzerland

^4^Université Clermont-Auvergne (UCA), Geolab, UMR 6042 CNRS, 4 rue Ledru, 63057 Clermont-Ferrand, France

^5^Dendrolab.ch, Department of Earth Sciences, University of Geneva, Rue des Maraîchers 13, 1205 Geneva, Switzerland

^6^Department F.-A. Forel for Environmental and Aquatic Sciences, University of Geneva, 66 Bvd Carl Vogt, 1205 Geneva, Switzerland

^7^V.N. Sukachev Institute of Forest SB RAS, Federal Research Center “Krasnoyarsk Science Center SB RAS” 50/28 Akademgorodok, Krasnoyarsk, 660036, Russian Federation

*Corresponding author: Olga V. Churakova (Sidorova), E-Mail: ochurakova@sfu-kras.ru

**SUPPLEMENTARY MATERIAL**

**a)**

**
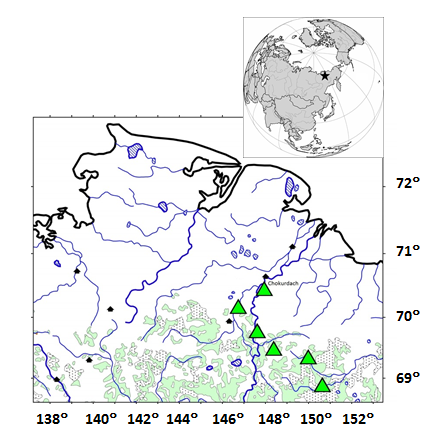
b)**


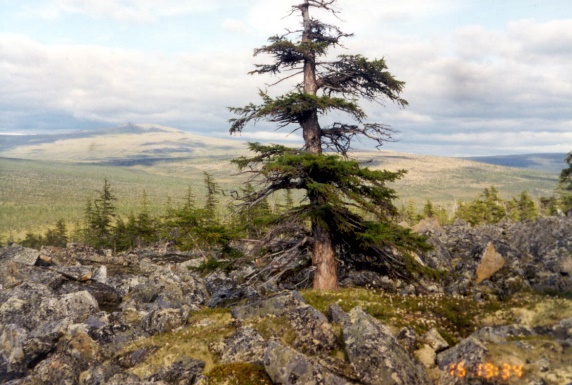


**c)**


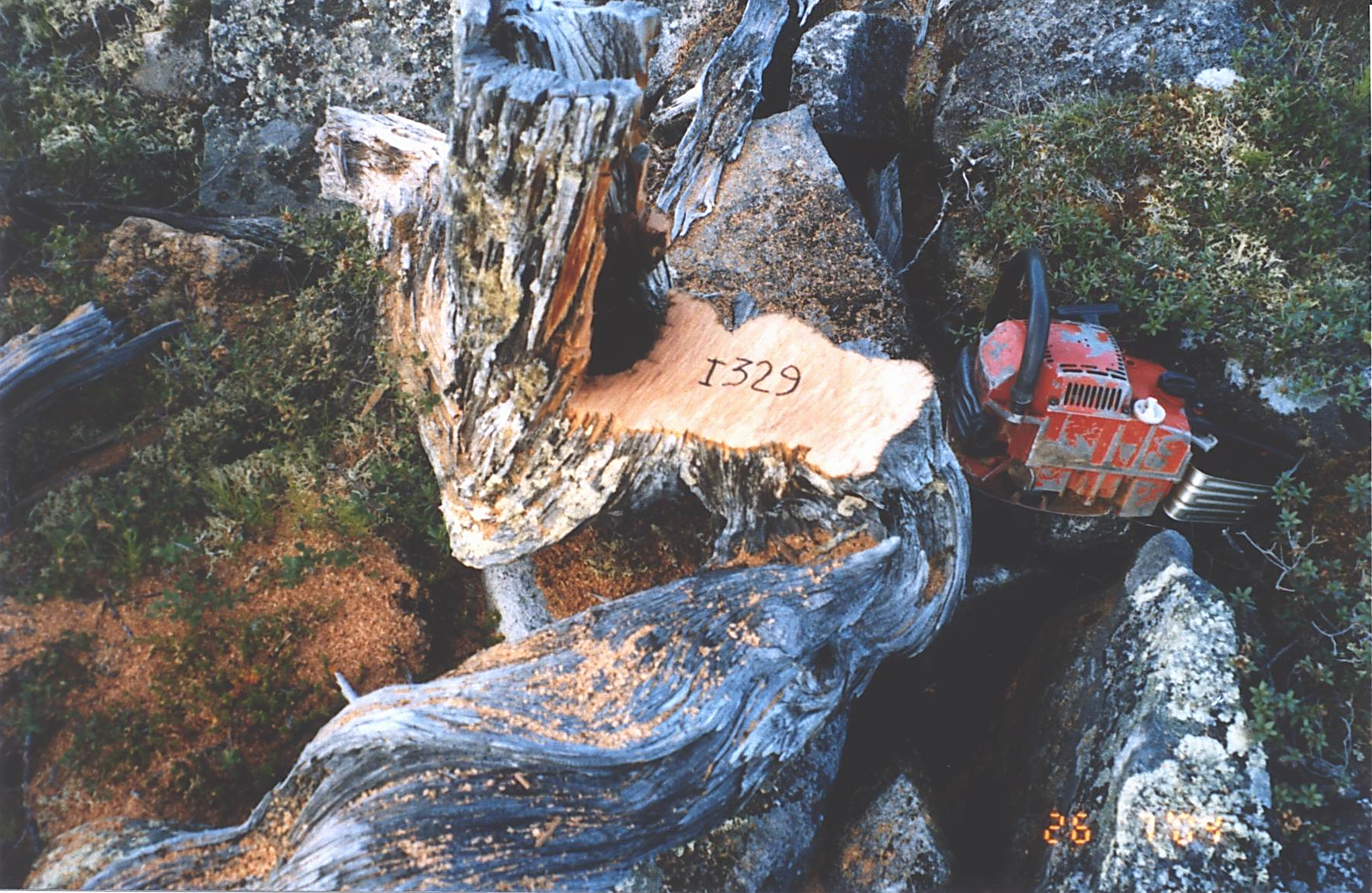


**Fig. S1.** Location of the study site in northeastern Yakutia (YAK) **(a)** and sampling plots for selection of relict larch wood and tree cores from living larch trees (*Larix cajanderi* Mayr.), which can reach 838 years old (CE 527-1364) **(b)**, while the dead wood trunks preserved on the permafrost surface can reach 1029 years old (CE 162-1191) **(c)** at the study site^26^. Photos made by M.M. Naurzbaev.

Map was created by using ESRI ArcGIS v.9 Software.

**
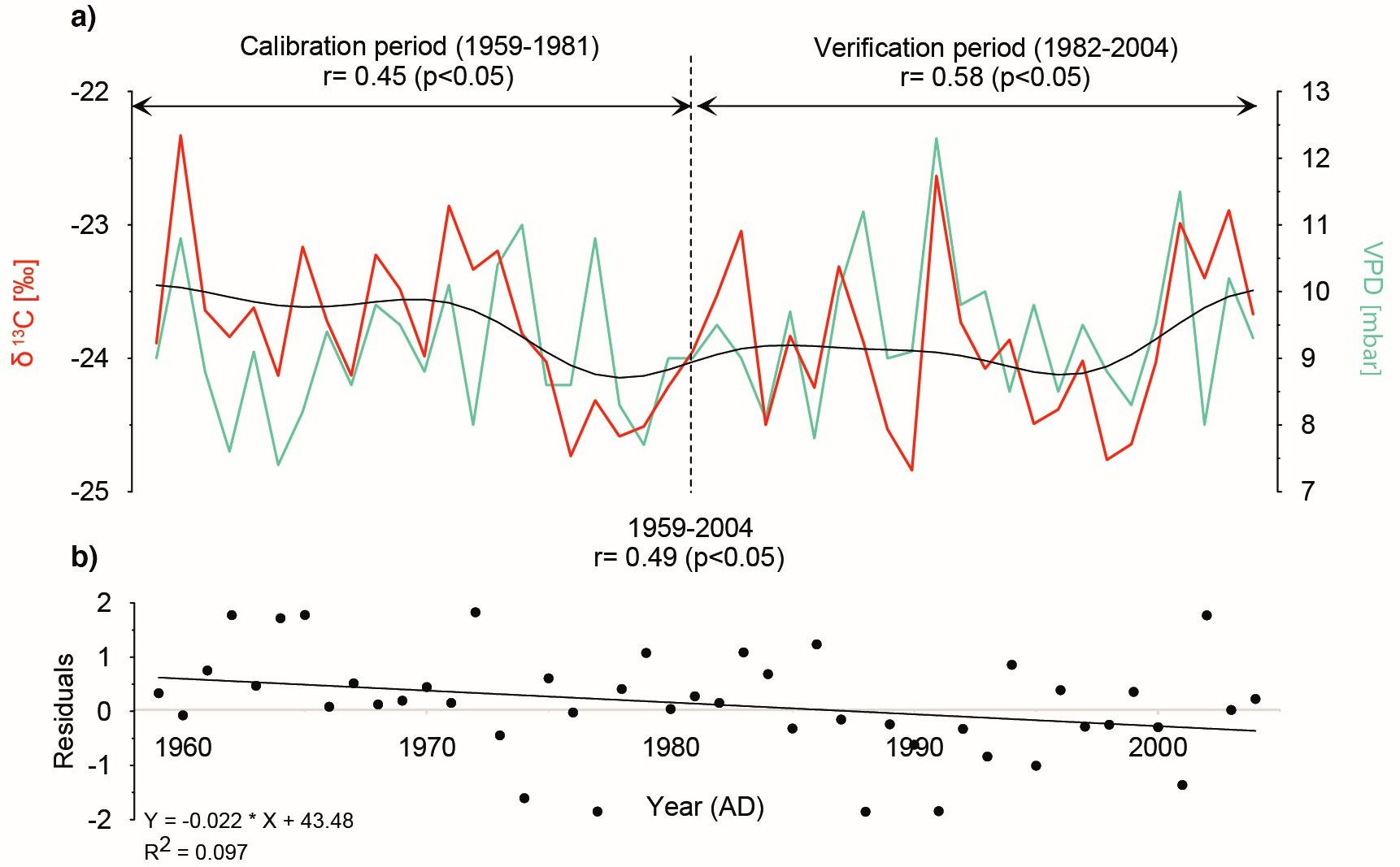
**

**Fig. S2.** Regression analysis of δ^13^C values versus instrumental vapor pressure deficit (VPD) data as the result of calibration (1959-1981) and verification (1982-2004) models **(a)**; The δ^13^C residual chronology is presented as the difference between observed and simulated VPD values for the period from 1959 to 2004 **(b)**.

**Table S1.** Identification number of samples used for the development of the stable carbon isotope chronology in larch (*Larix cajanderi* Mayr.) tree-ring cellulose from northeastern Yakutia (C-IND).

| N | Sample ID number | Pith/No pith (NP) | Height, m | First year | Last year | Age |
| --- | --- | --- | --- | --- | --- | --- |
| 1 | I212MEAN | P | 0.1 | 110 | 698 | 589 |
| 2 | I235A3DD | P | 0.1 | 528 | 853 | 326 |
| 3 | I301MED | P | 0.2 | 456 | 631 | 176 |
| 4 | I304MEDD | NP | 0.5 | 527 | 1364 | 838 |
| 5 | I305MED | NP | 0.1 | 464 | 652 | 189 |
| 6 | I308AFF | P | 0.5 | 1114 | 1493 | 380 |
| 7 | I315ACFF | P | 0.2 | 869 | 1320 | 452 |
| 8 | I319AOFF | NP | 0 | 1102 | 1233 | 132 |
| 9 | I320ADF | P | 0.5 | 767 | 1073 | 307 |
| 10 | I321AD | P | 0.4 | 770 | 1056 | 287 |
| 11 | I322AD | NP | 0.2 | 746 | 967 | 222 |
| 12 | I324AAFF | NP | 0 | 388 | 624 | 237 |
| 13 | I329CD | NP | 0.1 | 162 | 1191 | 1029 |
| 14 | IND296DF | NP | 0.2 | 1284 | 1520 | 237 |
| 15 | IND298DD | P | 0.2 | 1265 | 1642 | 378 |
| 16 | I333AAFF | NP | 1 | 1073 | 1588 | 516 |
| 17 | I334AD | NP | 1.5 | 61 | 388 | 328 |
| 18 | I335ADF | P | 0.9 | 708 | 1386 | 679 |
| 19 | I340AFF | P | 1 | 1496 | 1998 | 503 |
| 20 | I3422 | NP | 0.3 | 504 | 642 | 139 |
| 21 | I403AD | P | 0 | 1347 | 1608 | 262 |
| 22 | I42607AD | P | 0.6 | 890 | 960 | 71 |
| 23 | I433D | P | 0.6 | 1115 | 1329 | 215 |
| 24 | I426d | NP | 0.2 | 841 | 1011 | 171 |
| 25 | I478D | P | 0 | 825 | 1071 | 247 |
| 26 | I47903AD | P | 0 | 813 | 1077 | 265 |
| 27 | I497BD | P | 0 | 872 | 1056 | 185 |
| 28 | I483DF | NP | 1.5 | 1034 | 1344 | 311 |
| 29 | I48403AF | P | 1.4 | 850 | 1482 | 633 |
| 30 | I49001AF | P | 0.1 | 399 | 774 | 376 |
| 31 | I504 | NP | 1.3 | 1810 | 2004 | 195 |
| 32 | I518AD | NP | 0 | 1402 | 1562 | 161 |
| 33 | I552ABD | P | 0.5 | 1537 | 1640 | 104 |
| 34 | I56001AF | P | 0.3 | 338 | 795 | 458 |
| 35 | I58403AB | P | 0.7 | 873 | 1628 | 756 |
| 36 | I207AD | NP | 0.6 | 1091 | 1325 | 235 |
| 37 | I234DF | P | 0 | 1090 | 1263 | 174 |
| 38 | I241AB | NP | 0.6 | 1127 | 1808 | 682 |
| 39 | I249DD | P | 0.1 | 1082 | 1456 | 375 |
| 40 | I255DD | P | 1.3 | 990 | 1729 | 740 |
| 41 | I284DD | P | 0 | 1250 | 1470 | 221 |
| 42 | I286FF | NP | 1.3 | 1156 | 1387 | 232 |
| 43 | I290AD | P | 0.1 | 926 | 1065 | 140 |
| 44 | I299A | P | 0.1 | 1335 | 1623 | 289 |
| 45 | KMAS3_5D | P | 1.3 | 1552 | 2004 | 453 |
| 46 | KMAS2-3 | P | 1.3 | 1915 | 2004 | 89 |
| 47 | KMAS4-3 | P | 1.3 | 1882 | 2004 | 122 |
| 48 | KMAS3-5 | P | 1.3 | 1728 | 2004 | 276 |

**Table S2.** Statistical values for the calibration and verification periods between weather station July vapor pressure deficit data and the δ^13^C in cellulose.

| **Calibration** | | | | | | | **Verification** | | | | | |
| --- | --- | --- | --- | --- | --- | --- | --- | --- | --- | --- | --- | --- |
| Period | R | R^2^ | F-test | DW | RE | CE | Period | r | r^2^ | K_s_ | RE | CE |
| 1959-2004 | 0.49 | 0.24 | F=14.17 df=1.44  *P<0.001* | 1.82 |  |  | 1959-2004 |  |  |  |  |  |
| 1959-1981 | 0.58 | 0.34 | F=10.78 df=1.21  *P<0.001* | 2.06 |  |  | 1981-2004 | 0.45 | 0.20 | 0.42 |  |  |
| 1982-2004 | 0.45 | 0.20 | F=5.06 df=1.20  *P<0.001* | 1.81 | 0.49  *P<0.01* | 0.32  *P<0.01* | 1959-1981 | 0.58 | 0.34 | 0.58 | 0.18  *P<0.01* | 0.06  *P<0.01* |

**Table S3.** Summary table of the statistical characteristics for: a) 30 years before and after the shift, observed in the C-IND; b) the original (‰) and normalized (z-score) simulated July Vapor Pressure Deficit (VPD) data for the specific periods over the past 1489 years: Early Medieval Period (EMP) CE 516-799, Medieval Climate Anomaly (MCA) 800-1070, Little Ice Age (LIA) 1450-1850, Recent Period (RP) 1870-2004 and 1950-2004.

a)

| **Parameter** | **30-years before and after the shift*** | | | | | |
| --- | --- | --- | --- | --- | --- | --- |
|  | 750-779 | 780-810* | 810-840* | 1030-1060 | 1060-1090* | 1090-1100 |
| Mean | -24.64 | -24.30 | -23.35 | -23.57 | -24.48 | -24.81 |
| Median | -24.61 | -24.39 | -23.35 | -23.63 | -24.16 | -24.76 |
| SD | 0.21 | 0.62 | 0.64 | 0.56 | 0.91 | 0.54 |
| Min | -25.07 | -25.01 | -24.50 | -24.90 | -25.9 | -26.13 |
| Max | -24.2 | -22.34 | -21.90 | -21.81 | -22.74 | -23.60 |
| 25th% | -24.8 | -24.8 | -23.79 | -23.90 | -25.3 | -25.14 |
| 75th% | -24.4 | -24.07 | -23.02 | -23.28 | -23.73 | -24.45 |

b)

| Statistical parameters | **Period** | | | | |
| --- | --- | --- | --- | --- | --- |
|  | EMP  (516-799) | MCA  (800-1070) | LIA  (1450-1850) | RP | |
|  |  |  |  | (1870-2004) | (1950-2004) |
| Mean | -24.54 | -23.98 | -25.03 | -24.19 | -23.90 |
| Median | -24.63 | -23.97 | -25.05 | -24.21 | -23.88 |
| SD | 0.5 | 0.64 | 0.8 | 0.69 | 0.66 |
| N | 282 | 271 | 401 | 135 | 55 |
| Min | -26.05 | -25.31 | -27.49 | -25.85 | -25.86 |
| Max | -22.50 | -21.82 | -22.79 | -22.33 | -22.34 |
| 25th% | -24.87 | -24.41 | -25.51 | -24.70 | -24.38 |
| 75th% | -24.33 | -23.56 | -24.61 | -23.71 | -23.48 |

**Table S4**. Statistical characteristics of the z-score July VPD reconstruction for each 100-year time window and for the whole period.

| Statistical parameters | 516-615 | 616-715 | 716-815 | 816-915 | 916-1015 | 1016-1115 | 1116-1215 | 1216-1315 | 1316-1415 | 1416-1515 | 1516-1615 | 1616-1715 | 1716-1815 | 1816-1915 | 1916-2004 | 516-2004 |
| --- | --- | --- | --- | --- | --- | --- | --- | --- | --- | --- | --- | --- | --- | --- | --- | --- |
| Mean | 0.48 | 0.01 | 0.27 | 1.00 | 0.53 | 0.50 | -0.56 | -0.35 | -0.63 | -0.90 | -0.60 | 0.37 | -0.60 | -0.20 | 0.76 | 0.00 |
| SD | 0.89 | 0.43 | 0.70 | 0.77 | 0.68 | 1.01 | 0.71 | 0.97 | 0.94 | 0.91 | 0.92 | 0.90 | 0.52 | 0.91 | 0.81 | 1.00 |
| Min | -1.66 | -0.92 | -0.57 | -0.51 | -0.76 | -1.77 | -2.75 | -2.88 | -2.88 | -2.76 | -3.44 | -1.53 | -2.29 | -2.09 | -1.42 | -3.44 |
| Max | 2.62 | 1.45 | 3.38 | 3.34 | 2.01 | 3.53 | 1.48 | 2.04 | 1.51 | 1.79 | 1.22 | 2.33 | 0.54 | 1.61 | 2.90 | 3.53 |
| 25th% | -0.20 | -0.28 | -0.15 | 0.41 | 0.02 | -0.26 | -1.06 | -0.98 | -1.29 | -1.52 | -1.19 | -0.32 | -0.87 | -0.86 | 0.06 | -0.60 |
| 75th% | 1.17 | 0.31 | 0.45 | 1.54 | 1.07 | 1.20 | -0.14 | 0.16 | 0.06 | -0.32 | 0.07 | 1.03 | -0.30 | 0.51 | 1.31 | 0.65 |
